# The benefits of digitisation of psychiatric care facilities

**DOI:** 10.1192/bji.2023.16

**Published:** 2023-08

**Authors:** David Skuse

**Affiliations:** Professor of Behavioural and Brain Sciences, Division of Population, Policy and Practice, UCL Great Ormond Street Institute of Child Health, London, UK. Email d.skuse@ucl.ac.uk

**Keywords:** Telehealth, rural populations, Pakistan, eLearning, flooding

## Abstract

The potential benefits of providing digital mental healthcare to isolated rural populations are emphasised in two articles from Pakistan. Novel programmes of support have been instituted by both private and publicly funded services.

Over the past decade there has been increasing emphasis on the potential benefits of integrated healthcare, across the lifespan. In response, the World Health Organization (WHO) initiated a programme of universal health coverage (UHC), which aimed to provide the human right to health (including mental health) to everyone, especially those currently underserved populations in low- and middle-income countries. The delivery of essential health services to more than one billion people was a 2022 WHO strategic goal.

## The Sehat Sahulat Program and AKDN Digital Health Programme

In Pakistan, there has historically been a paucity of public health facilities, with limited access and quality of care especially in rural areas. To address this problem, in 2015 the provincial government of the Khyber Pakhtunkhwa (KP) introduced the Sehat Sahulat Program (the Health Facility Program) under the UHC initiative. On receipt of a special card, families could purchase treatment up to a certain value, each year, from several hundred public and private health facilities in the region. The programme is subsidised by the government, which pays a premium per eligible family to the State Life Insurance Corporation for its administration. Over the past few years, this programme has been extended to other provinces.

Linked to this innovation, the Aga Khan Development Network (AKDN) Digital Health Programme is also trying to address the problem of providing health services to communities living in remote and isolated regions of South-Central Asia and East Africa. In many of those areas, families seeking quality specialist healthcare, including mental healthcare, must travel to distant cities. They are burdened with unnecessary expenses and travel time. The AKDN owns over 400 healthcare facilities in Pakistan and is using public–private partnerships in some regions. Through a digital network, patients receive a variety of teleconsultation services, including diagnostic services, and clinicians benefit from eLearning services to help reduce professional isolation for those working in remote areas.

## This issue

In this issue, we have two papers on mental health in Pakistan. Alvi et al^1^ consider the economic costs of providing services in general, specifically mentioning the Sehat Sahulat Program, which now covers consultations for that purpose, but to a limited extent. Riaz et al (2023)^2^ discuss the 2022 floods, which had a massive impact on several provinces. In the short term, this disruption led to a risk of infectious diseases, but in the longer term the impact will put a considerable strain on the minimal services available to provide psychosocial support.
